# Improving the well-being of children and youths: a randomized multicomponent, school-based, physical activity intervention

**DOI:** 10.1186/s12889-016-3794-2

**Published:** 2016-10-28

**Authors:** Søren Smedegaard, Lars Breum Christiansen, Pernille Lund-Cramer, Thomas Bredahl, Thomas Skovgaard

**Affiliations:** 1 Research and Innovation Centre for Human Movement and Learning, Odense, Denmark; 2 University of Southern Denmark, Campusvej 55, 5230 Odense M, Denmark; 3 Department of Sports Science and Clinical Biomechanic, Campusvej 55, 5230 Odense M, Denmark; 4 University College Lillebaelt, UCL Campus Odense, Niels Bohrs Allé 1, 5230 Odense M, Denmark

**Keywords:** Well-being, School, Children, Youth, Physical activity, Physical education, Denmark

## Abstract

**Background:**

The benefits of physical activity for the mental health and well-being of children and young people are well-established. Increased physical activity during school hours is associated with better physical, psychological and social health and well‐being. Unfortunately many children and young people exercise insufficiently to benefit from positive factors like well-being.

The main aim of this study is to develop, implement and evaluate a multi-component, school-based, physical activity intervention to improve psychosocial well-being among school-aged children and youths from the 4^th^ to the 6^th^ grade (10–13 years).

**Methods:**

A four-phased intervention – design, pilot, RCT, evaluation - is carried out for the development, implementation and evaluation of the intervention which are guided by *The Medical Research Council* framework for the development of complex interventions. 24 schools have been randomized and the total study population consists of 3124 children (baseline), who are followed over a period of 9 months. Outcome measure data at the pupil level are collected using an online questionnaire at baseline and at follow-up, 9 months later with instruments for measuring primary (general physical self-worth) and secondary outcomes (self-perceived sport competences, body attractiveness, scholastic competences, social competences and global self-worth; enjoyment of PA; self-efficacy; and general well-being) that are both valid and manageable in setting-based research. The RE-AIM framework is applied as an overall instrument to guide the evaluation.

**Discussion:**

The intervention focuses on the mental benefits of physical activity at school, which has been a rather neglected theme in health promotion research during recent decades. This is unfortunate as mental health has been proclaimed as one of the most important health concerns of the 21^st^ century. Applying a cluster RCT study design, evaluating the real-world effectiveness of the intervention, this study is one of the largest physical activity intervention projects promoting psychosocial well-being among children and youths. Through a comprehensive effectiveness evaluation and a similar substantial process evaluation, this study is designed to gain knowledge on a broad variety of implementation issues and give detailed information on project delivery and challenges at the school level – among other things to better inform future practice.

**Trial registration:**

Date of registration: 24 April 2015 retrospectively registered at Current Controlled Trials with study ID ISRCTN12496336

## Background

The benefits of physical activity (PA) for the mental health and well-being of children and youths are well-established. Among other things, regular PA can help build social skills and self-esteem – the latter commonly viewed as a key indicator of positive well-being [1–4].

Unfortunately many children and young people exercise insufficiently to benefit from positive factors like the ones mentioned above [5, 6]. In recent years, a number of large school-based interventions have been conducted, in the effort to increase physical activity in larger, blended populations of children [7–14], as well as interventions aiming at children’s mental health and well-being [3, 15, 16]. An overall conclusion of these studies is that increased physical activity during school hours is associated with better physical, psychological and social health and well‐being.

A review on the effectiveness of school-based, physical activity interventions found significant, albeit varied, effects on self-concept and attitudes towards exercise [17]. A comprehensive review of reviews, by Biddle and Asare [1], substantiates the claim that PA has an overall positive effect on mental health and well-being. A few years ago, Bailey and colleagues summed up the issue and stated: *‘There is compelling evidence that regular PA can have a positive effect on emotional well-being, especially for children and young people’* [18]. At the same time, Bailey and colleagues underlined that there is no “automatism” regarding the connection. PA’s contribution to well-being is conditional to the context and especially the social climate generated by e.g. educators [18–20]. Positive experiences with PA form part of a “virtuous cycle” and improve self-concepts and even more, overall well-being, while negative experiences transform the relationship to a “vicious cycle” through which the person becomes more and more disaffected in relation to PA [18].

### The school as the setting for PA interventions

In many countries, school-based approaches to PA have the obvious advantage that the children who need it most are fairly reachable, due to the fact that the vast majority of children and adolescents receive their primary education in public schools. Furthermore, health and well-being is an integrated part of the public school curriculum, which means that there are qualified educators and an existing culture for teaching and learning activities related to health, well-being and PA [21].

**Fig. 1:**
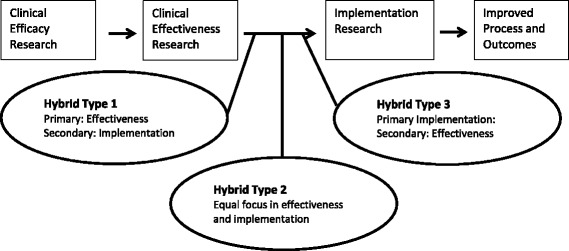
Hybrid-effectiveness continuum. Hybrid effectiveness-implementation designs as part of the clinical research continuum, adapted from [58]

Research on the effectiveness of school-based physical activity interventions and other health interventions points out a number of methodological issues worth mentioning [17]. First of all, development of the intervention should be based on best available evidence and should be thoroughly pilot tested in settings similar to the actual intervention environments. Secondly, program fidelity - meaning the degree to which an intervention is implemented as intended – should be monitored and reported on. Tracking implementation fidelity is essential to understanding the program impact. Especially for the latter reason, intervention research should incorporate systematic evaluations on both the process and the effectiveness of school-based, physical activity intervention [17], hence known as hybrid type 2 studies (see Fig. 1).

Furthermore, a theoretical approach in intervention science is needed [22]. Theories dealing with both intervention processes and effectiveness contribute to explaining how and why certain outcomes are achieved; identifying core elements that influence implementation; distinguishing between program theory failure and program implementation failure and, finally, in further program development [22].

Taking these issues into account, the aim of this article is to present the development, design and main components of the *Move for Well-being in Schools (MWS)* intervention study.

## Methods and design

### Study aim and overview

The main aim of MWS is to develop, implement and evaluate a multi-component, school-based, physical activity intervention to improve psychosocial well-being among school-aged children and youths from the 4^th^ to the 6^th^ grade (age 10–13 in the Danish school system). The study has a special focus on the group of children who are most at risk of experiencing a ‘vicious cycle’ in physical activity at school, and thus could possess lower motivation and decreased self-confidence for engaging in normal PA activities in school.

**Fig. 2:**
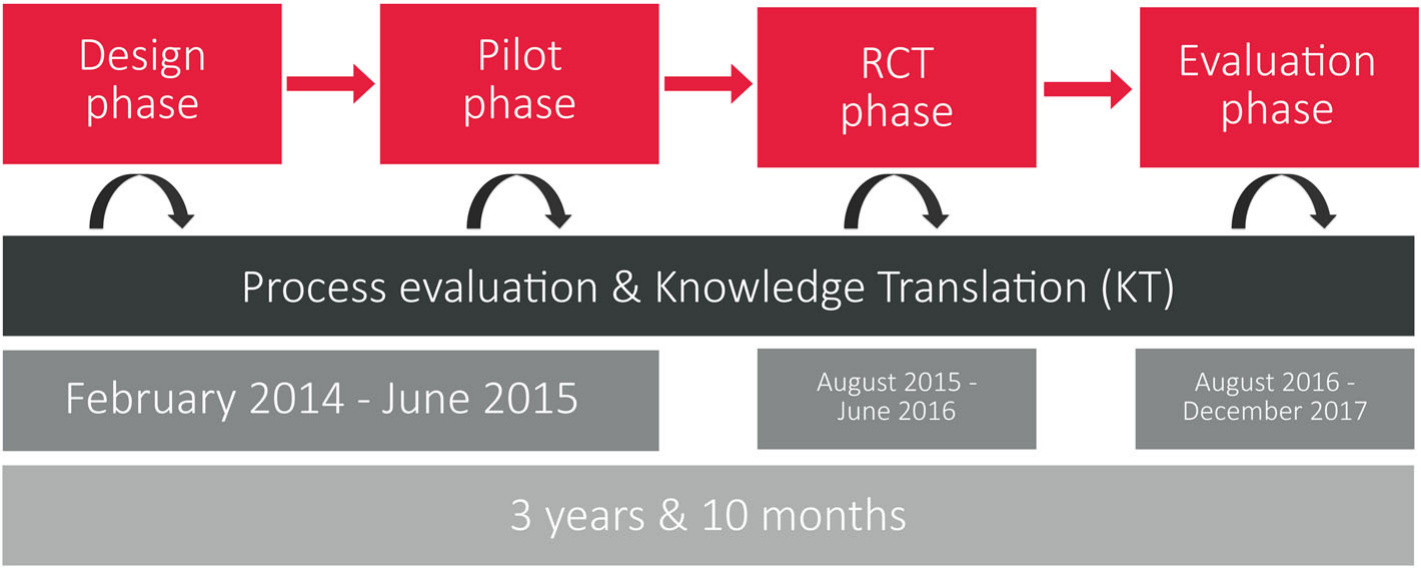
Study design. A four-phased intervention. Overview of the study period

The development, implementation and evaluation of the intervention are guided by *The Medical Research Council* framework for the development of complex interventions. Since it was first published in 2000, the framework has been widely used to identify and tailor activities for selected target audiences [23]. For this study, a four-phased intervention is carried out in the years 2014–2017 (Fig. 2). The four phases consist of a *design phase*, *pilot testing phase*, *randomized controlled trial phase* and *a program evaluation phase*.

Knowledge Translation (KT) is a key activity in all stages of the intervention (Fig. 2.). KT is understood as a dynamic process that includes synthesis, exchange and application of knowledge to improve health and well-being and provide more effective services [24]. For a number of years, it has been stressed that such processes must ensure the combination of best available research evidence and local contextual knowledge by facilitating close interaction between researchers, end users and other relevant stakeholders [25, 26].

### Design phase

This phase started with a scoping review summarizing published knowledge on both implementation and effectiveness of similar interventions. Furthermore, a number of structured group interviews were conducted with members of the target group in order to get further, contextual insight with regard to the needs, wishes, challenges and perceived qualities formulated by pupils aged 10–13 in relation to engaging in and enjoying physical activity. Informed by findings from the mentioned materials, a preliminary intervention program was qualified via four workshops in which researchers, school managers, teachers, pedagogues, and organizations dealing with school sport and physical education participated. The stakeholders constituted the Project Development Group (PDG). The main aim was to incorporate professional and/or practice-based expertise and knowledge into the further development of the final intervention.

The design phase consolidated the project’s theory of change and facilitated close interaction between researchers, end users and other relevant stakeholders in connection with intervention development. Thus, besides guiding the intervention model, the design phase contributed to continuous, broad-based stakeholder involvement. Evidence suggests that evaluations and key intervention findings are more likely to be used on a long-term basis if stakeholders are involved in and committed to the program and assessment processes. Thus, it has been shown that co‐creation, where research users are directly involved in the research process, facilitates sustained and creative use of research results [27, 28].

The output of the design phase was a comprehensive intervention program based on best available evidence, close collaboration with stakeholders and grounded in a solid theoretical approach, in particular to the area of motivation – as construed by Edward Deci and Richard Ryan in their self-determination theory (SDT) [29]. According to Deci and Ryan, the main architects of SDT, human motivation is essentially based on three innate psychological needs: competence, autonomy and relatedness. The SDT theory’s distinction between self-determined or autonomous and controlled types of motivation reflects individual reasons for participating in activities. The PA in this particular intervention program is therefore designed to meet these three psychological needs in order to motivate all pupils [29, 30].

The intervention program itself consists of three components targeting four settings for school-based physical activity (Table 1). Basically, the intervention program strives to improve the activities conducted and the social and pedagogical climate in which they are performed. For this reason, the practitioners were equipped with a Tailored Activity Program (TAP), including educational materials, planning guides and course plans for incorporating PA throughout the school day. The materials were both delivered in printed versions and also made available on the intervention website, which also contained overall information on the program goals and deliverables.

**Table 1:** Intervention program components. Overview of the intervention program, what it contains and the keyword that describes the components

Intervention program	Part	What	Keywords
Components	Competence Development Program (CDP)	4 Workshop days Vodcast – short video podcast with info and themes regarding the program. Inspirational day at the schools – “kick off – day”	Focusing on underlying theory of PA intervention Trying out core activities Knowledge translation Inspiration
	Tailored Activity Program (TAP)	Website Educational materials, planning guides and course plans	Availability Support Inspiration
	Coordination Group (CG)	A representative from 4th, 5th, and 6th grade and from school management	Connection between educators and management Supplying support Contact to research team Two supervision visits at each school
PA settings	Physical education classes	6 PE courses each of 6 × 90 min where 2 of these are mandatory and the last 4 can be chosen from a variety of lesson plans	8 different courses Team based Student involvement Competence focus
	In-class activities	2 activity breaks per day/50 mins per week	In-class activities/pause activities Differentiate purpose: social, energy, relax and coordination
	Recess activities	3 sessions per week, average 30 min per session	Initiation and support of recess PA
	Theme days	2-3 theme days arranged over the school year	Development of activities with pupils. Focus on well-being at school.

The TAP was supported by a Competency Development Program (CDP) for educators, focusing on the underlying theoretical approach and opportunities to try out core activities in practice. Furthermore, each school was supported in setting up a Coordination Group (CG), which received support via biweekly information e-mails, two local supervision visits with the research team during the intervention period and the possibility to contact the research team throughout the period by mail or phone. The four selected settings were: Physical education classes, in-class activities, recess activities and theme days.

### Pilot phase

*The pilot phase* included a structured assessment of whether the initial intervention program could, in fact, be implemented and adapted to the school setting. This entailed systematic appraisals of the acceptability and feasibility of delivering the intervention [23]. The pilot phase was divided into a *preparation phase* that lasted three months and an *action phase* of four months, where the actual intervention was piloted at five schools.

In the *preparation phase*, the Competency Development Program (CDP) provided enrolled educators with knowledge and skills tailored to carry out differentiated instruction and teaching activities, with the aim of supporting pupil motivation for and engagement in school-based PA. A specific focus was to make sure that educators acquired further skills and competencies to engage with a diversified group of pupils, displaying a large variation in performance levels, and to provide additional support to pupils who, for various reasons, were less motivated to engage in school-based PA.

*The action phase* was conducted at the same five schools that initially delivered members to the above-mentioned Project Development Group. In this way, stakeholders taking part in the *design phase* were given the opportunity to continue their involvement and, importantly, to secure further development via active feedback on effectiveness, implementation and feasibility issues.

### Adjustment of the intervention program

*Pupil level* assessment of the pilot action phase was conducted via qualitative focus group interviews, including two boys and two girls from each grade (4^th^ to 6^th^ grade) in three out of five pilot schools. The interviews lasted 30 min and were semi-structured. The pupils were encouraged to engage in joint dialogue on how best, on the one hand, to address perceived problems and deficiencies and, on the other hand, to strengthen key qualities of the various PA intervention components [31].

*School level* assessments of the pilot action phase were based on face-to-face evaluation meetings with the practitioners and managers involved in the pilot intervention and on questionnaires distributed to them. The overall aim was to gather information on the participants’ evaluation of the relevance and impact of the interventions in relation to promoting PA and well-being in the school setting. The primary target group was the educators taking part in the aforementioned CDP. Interviews with school management and the CG were performed midway through the pilot phase and at the end of the pilot phase. In a fourth and final workshop, findings from pupil and school level assessments were discussed with the CG from the pilot schools.

The pilot phase led to a number of smaller intervention improvements, as well as an increased focus on securing a durable intervention delivery system. Furthermore, specific modifications were made to the CDP and TAP. An example of a modification was omitting an intended e-learning component through vodcasts (video podcasts) appertaining to the teacher study groups at the schools. Due to time constraints, the educators were not able to engage with the vodcasts in the study groups, and the themes were instead incorporated into workshop sessions and other materials. Another modification example is the length of each PE course (cf. Table 1), which was reduced from six to four 90 min lessons. The reason for that was that teachers voiced challenges with motivating pupils to fully engage with the same theme in six consecutive PE lessons.

### RCT phase

*The randomized controlled trial (*RCT) entails the implementation of the final intervention program (Table 1) in the selected settings. Based on statistical power calculations and the capacity of the research set-up, a target of 12 schools was set for both the control and the intervention group. Municipalities were selected based on maximizing geographic spread, difference in size and difference in allocated budget for public schools [32]. Municipalities were not contacted if they already had large scale school development projects; were located near universities or university colleges other than the operating unit (University of Southern Denmark); located in relatively hard to reach locations; or had less than 6 schools in the entire school district. Based on initial screening, 11 of 98 Danish municipalities were contacted. After consent, local authorities initiated the first contact with schools or gave the research team permission to contact the schools directly. The research team held individual meetings with all interested schools and a total of 24 schools were enrolled. A stratified randomization was then conducted with three strata and with the constraint of an even distribution of schools from each municipality in the intervention group and the control group respectively. The three strata were defined by school typology, based on school size and district socio-economic status. Stratum A: Small schools, <70 pupils in grades 4–6, often located in small rural villages; Stratum B: Larger schools, >70 pupils, with low socio-economic status; and Stratum C: Larger schools with high socio-economic status. Beginning with Stratum A, schools were randomly assigned to either intervention or comparison by means of simple randomization - while adhering to the constraint of even distribution of schools from the various municipalities in the intervention and control group.

**Fig. 3:**
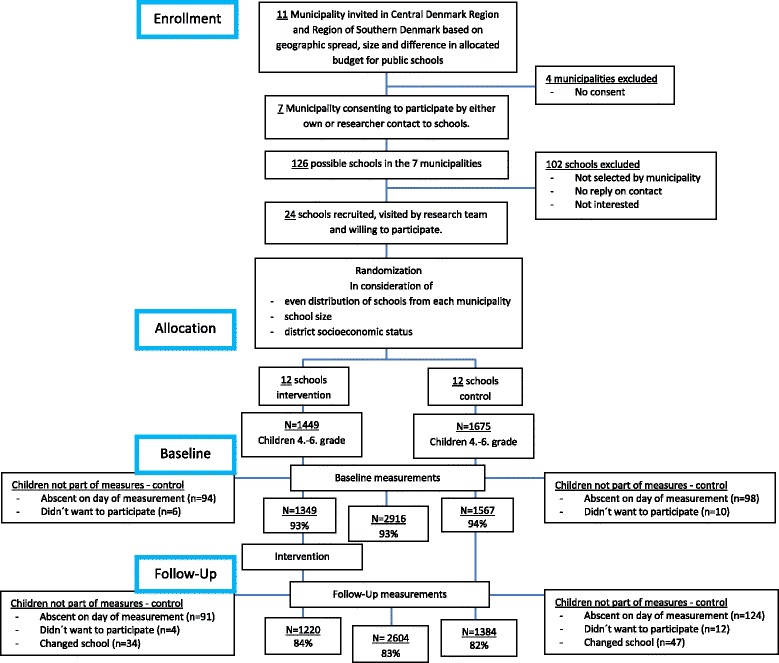
Flowchart of recruitment, randomization and baseline questionnaire

The initial part of the RCT phase is illustrated in Fig. 3.

### Program evaluation

*The program evaluation phase* examines the final intervention program with a focus on both effectiveness and implementation: Did the intervention go as planned; what actually happened; and was the intervention effective in improving the psychosocial well-being of the target population? In addition to the effectiveness issue, the program evaluation provides insight on how the intervention was delivered, what barriers and opportunities it created for teachers, and how the intervention was experienced by the pupils. A mixed-method evaluation design, combining qualitative and quantitative approaches, is applied to gather valid information on both effectiveness and implementation issues. The RE-AIM framework is applied to ensure a holistic assessment of intervention impacts [33]. RE-AIM has been widely used to enhance the quality, speed, and public health impact of efforts to translate research into practice [33]. The framework is made up of five key elements - Reach, Effectiveness, Adaptation, Implementation and Maintenance.

### Outcome measures

The design and pilot phase also comprised testing, selecting and adapting instruments for measuring primary (general physical self-worth) and secondary outcomes (self-perceived sport competences, body attractiveness, scholastic competences, social competences and global self-worth; enjoyment of PA; self-efficacy; and general well-being) that were both valid, reliable and manageable in setting-based research.

The selected survey instruments have systematically been translated and adapted in order to secure Danish versions that are conceptually equivalent to the original English instruments (if not already available in Danish). The procedure used in this study is guided by the WHO^1^ *Process of translation and adaptation of instruments.* An initial forward translation from English to Danish was conducted by a professional translator familiar with the terminology of themes covered by the instruments, followed by a cross-cultural adaptation. A second professional translator conducted a back translation of the Danish instruments to English. Two bilingual experts compared the original and back-translated English versions of the survey instruments. The experts discussed their findings and suggestions with the research team, supplemented by other content resource people, which led to minor revisions [34].

In connection with the pilot phase, the compiled survey instrument was tested by groups of pupils followed by a cognitive validation process. Questionnaire characteristics - e.g. completion time and numbers of questions, and repeated clarifying questions on specific topics - were evaluated. The cognitive validation consisted of interviews with selected pupils and focused on their understanding of the various survey items [31]. Based on the evaluation, the questions were modified to decrease reading load and completion time, and a short version of the the Physical Activity Enjoyment Scale (PACES) questions was chosen.

In the RCT phase, data at the pupil level are collected using an online questionnaire at baseline and at follow-up, 9 months later. The questionnaire is administered in a quiet setting during school hours. A research assistant and an educator are present during data collection to ensure independent and confidential responding, to provide verbal information on how to respond to items and to answer possible questions from the pupils. The questionnaire design and purpose is described below.

#### Self-perception

Thirty six questions are used to measure participant’s self-perception. The Sport Competences, Body Attractiveness and Physical Self-worth subscales from the Children’s Physical Self-Perception Profile (C-PSPP) is used to assess changes in the primary outcome measures: Physical self-concept [35, 36]. The Scholastic Competence, Social Competence and Global Self-worth subscales from the Self-perception Profile for Children (SPPC) are included to measure aspects of psychosocial well-being. The SPPC, developed by Susan Harter, is widely used and found to be valid and reliable [37–39]. All scales in the SPPC and C-PSPP are calculated as the mean of 6 items. Each item consists of two statements, which are scored on a four-point scale using a structured alternative format designed to reduce social desirability. Firstly, the pupil is asked to decide what kind of kid he or she is most like in each statement. E.g. “Some kids do very well at all kinds of sports, BUT other kids don’t feel that they are very good when it comes to sports”. Next, the pupil has to decide whether the selected description is “Really True for Me” or “Sort of True for me”. A score of 1 indicates the lowest perceived competence or adequacy, and a score of 4 reflects the highest level of competence or adequacy.

#### Socio-demographics

Four structured questions are used to determine the socio-demographic characteristics of the pupils. Furthermore, family social class is measured based on information on parental occupation. The information is coded into occupational social class I-V, economically active, or unclassifiable (VI), economically inactive (VII), unclassifiable (VIII), missing information (IX). Family social class is equal to the parent with the highest occupation social class [40].

#### Physical activity

Leisure time physical activity is assessed using two structured questions. Firstly, pupils are asked to choose from four statements denoting different levels of leisure time PA (e.g. engaging in organized sports several times a week or preferring sedentary activities e.g. playing with the computer and listening to music) [41]. Secondly, the pupils are asked how often they are physically active at a level that makes them breathe hard and fast and/or gets them sweating. This item is scored on a 6-point Likert scale from “Everyday” through “Never”. The frequency of physical activity during recess is measured with a single question using a 4-point Likert format ranging from “Several times a day” to “Rarely or never”. Lastly, the pupils are required to answer how often they walk or bike to school on a 4-point Likert Scale (“Everyday” to “Never”).

#### Self-efficacy

Measured using 8 questions. The scale uses a single factor 5-point Likert format and is an adapted version of an 8-item questionnaire previously developed for use with 5^th^, 6^th^ and 8^th^-grade girls (PASES) [42–44]. In accordance with Bartholomew et al. [45], the original wording is changed from an explicit focus on *leisure time* physical activity, to encompass self-efficacy regarding PA in general (leisure and school time). The pupils are asked to select how much they agree with eight statements ranging from “Disagree a lot” to “Agree a lot”. Each item is scored from 1 to 5, with a score of 1 indicating lowest self-efficacy.

#### Enjoyment

Is measured using seven negatively worded questions from the Physical Activity Enjoyment Scale (PACES). The scale uses a 5-point Likert format and is an adapted version of the 16-item version of PACES. This instrument has been validated for use with children and adolescents [46–51]. Pupils are asked to select how much they agree with seven statements relating to different feelings about physical activity (“Agree a lot” to “Disagree a lot”). Each item is scored from 1 to 5, with a score of 1 indicating lowest enjoyment. E.g. “When I am physically active…I feel bored”.

#### Perception of physical education (PE)

Five structured questions are used to measure the perception of PE in relation to the frequency of participation and involvement in, the motivation for and the enjoyment of PE. Each question is scored on a 4 or 5-point Likert scale.

#### School and general well-being

Information relating to psychological well-being and school physical and psychosocial environment is measured using 13 questions stemming from the Danish national survey of well-being in the school-aged population. These questions are implemented to enable comparison not only within the study sample but also with national distributions on these key elements.

#### Health-related quality of life (HRQoL)

Information relating to the participant’s perception of general well-being is measured using the KIDSCREEN-27 Quality of Life Questionnaire for Children and Adolescents [52] - The KIDSCREEN Group Europe 2006). This tool consists of 27 items on physical well-being (5 items), psychological well-being (7 items), autonomy and parent relationship (7 items), peer and social support (5 item), and school environment (5 items). Each item is scored on a 5-point Likert scale with a timeframe of one week. Higher scores indicate better HRQoL, e.g. “Thinking about the last week…Have you felt lonely?”

### Evaluating implementation

This part of the program evaluation considers the adoption, implementation and short-term maintenance of the intervention program according to the RE-AIM framework. The focus is on three levels of operation: the strategic level (school managers and coordination group CG), the operational level (educators) and the user/beneficiary level (the pupils).

At the *user/beneficiary level,* the main objective is to assess pupils’ experiences with the intervention components. Did they experience a change, and did the change increase their motivation for and participation in physical activities at school? There is a specific focus on pupils who are most at risk of experiencing a ‘vicious cycle’ in relation to physical activity at school. Pupils at two schools are especially closely followed by a researcher using ethnographic methods, taking part in everyday school life. Informal interviews are conducted, which are supported by focus group interviews with pupils and individual interviews with educators. Approximately 25–30 days of observation, 10 focus group interviews and interviews with 4 educators are conducted at the two selected schools. To increase the information on the user-level, class evaluations are conducted at the end of the study in one random class at each grade level at all 24 schools. The class evaluation collects the immediate experience of school PA for pupils at all schools and focuses on the four settings for PA included in the intervention program (Table 1).

At the *operational level,* the main objective is to assess the fidelity and feasibility of the intervention. Did the educators receive enough information about the intervention and were they able to conduct activities as intended? The evaluation focus’ on self-efficacy in relation to executing activity components, perceived relevance and educators experienced resistance from the pupils regarding intervention participation. Three times during the intervention period, educators are asked to fill in online questionnaires on issues like these. Additionally, a selected group of PE teachers at four intervention schools are more intensively followed - focusing on their practice of the PE lessons designed for this particular intervention program (Table 1). Finally, the research group is informed about general progress, barriers and successes in connection with the previously mentioned supervision visits to each school. At the meetings, the researchers follow PE classes, observe recess and in-class activities and engage in informal conversations with the educators.

At the *strategic level,* the main objective is to assess the context in which the educators are operating. Did the school managers and CG support the intervention program, and did they ensure a collaborative effort to make it a whole-school approach? School managers are interviewed as regards their role in both leading and managing the intervention, and the *CGs* participate in two meetings with the research team during the supervision visits. The meetings center on four statements related to the role of the CG:

- *Well-being in school* is running fully as intended in our school
- Our colleagues are positive towards *Well-being in school*
- *Well-being in school* is a priority at our school and receives adequate support from the school management
- The three bearing SDT-principles - competence, autonomy and relatedness – are evident for all involved as leading principles of *Well-being in school*.

The statements are visually presented via analog continuum scales and the CGs are asked to state the level of implementation by placing a mark on scales for each of the four statements. The rest of the meeting concerns the reasons for the level of implementation, and how the CGs can improve the current status. The individual ratings from the members of the CGs are summarized into one ‘Room For Improvement’ (RFI) score for each school.

### Statistical considerations

Sampling is school-based (cluster randomized) and individual respondents are therefore not independent. Some of the variances in measurements can be ascribed to class, school and surrounding environment [32]. This internal variation at school and class level was accounted for in the sample size calculations and will be accounted for in the effect analyses.

Based on baseline data from the pilot study, an intracluster correlation coefficient (ICC) was calculated for the primary outcome (mean of ‘physical self-worth’ from the Children’s Physical Self-Perception Profile) using ‘clustersampsi’ in STATA version 14 [53]. With a mean of 3.12, an SD of 0.70, a significance level of 0.05, a statistical power of 0.80, an ICC of 0.025 at school-class level, 72 classes from 12 schools on each arm and approximately 20 pupils in each class, it will be possible to detect a difference of 0.09. The mean of ‘physical self-worth’ should, therefore, be increased from 3.12 to 3.21 before a significant effect can be detected.

The effect of the intervention is tested using multilevel models adjusted for relevant, potentially confounding factors (e.g. baseline outcome measure, gender, age and family social class).

When testing the overall effectiveness of the intervention, the primary outcome measures will be evaluated both as a continuous outcome and as a logistic outcome evaluating the proportion of students below a given threshold. All analyses will be performed according to the intention-to-treat principle, and supplemented with exploratory analyses to test associations between outcome measures and implementation fidelity using data from the process analyses. Because of the cluster structure of the data, random effects for school and class will be included in all analyses.

### Ethical considerations

In conducting research with human subjects, and especially children, ethical considerations should be highly prioritized - both in relation to the intervention and the evaluation thereof. PA interventions are often designed to change individual and/or collective views on the qualities of a physically active lifestyle. This could increase the pressure of living up to such active-living ideals and possibly cause adverse side effects, for example, eating disorders, excessive training and stigmatization of overweight individuals [54]. The current intervention is based on principles of an inclusive approach to PA in school, where students have positive experiences together with classmates. Still, it is possible that the interventions have adverse side effects and cause uncomfortable situations for some children. The likelihood of such unintended, negative side effects occurring is deemed as rather limited.

The main risk to the participants in the current study is potential embarrassment and disclosure of sensitive information to others [55]. The nature of some of the survey questions regarding self-perception and well-being could be regarded as sensitive, and some children could feel uncomfortable answering these. In particular, if the pupil has low self-perceptions or is struggling with bullying or exclusion in school, this could be an issue. Pupils were informed to complete the questionnaires individually and dividing cardboard was used between the tables to maximize privacy (for further information see *Ethics approval and consent to participate).*

## Discussion

Standing on the verge of the program evaluation phase, the developed school-based, physical activity intervention holds some promising strengths, as well as considerations. These will be summarized and discussed in the following section.

First of all, the intervention focuses on the mental benefits of physical activity at school, which has been a rather neglected theme in health promotion research during recent decades. This is unfortunate. Mental health has been proclaimed as one of the most important health concerns of the 21^st^ century, as stated early on by WHO in their 2004 summary report, *Promoting Mental Health*. On top of this the mutually dependent relationship between physical activity and mental health has not yet been fully uncovered [1, 56].

Another characteristic feature of the study is the cluster RCT study design, which evaluates the real-world effectiveness of an intervention. 24 schools were randomized and the total study population consists of 3124 children (baseline), who are followed over a period of 9 months. To our knowledge, this makes it one of the largest physical activity intervention projects promoting psychosocial well-being among children and youths.

Alongside the comprehensive effectiveness evaluation, the study is designed to gain knowledge on a broad variety of implementation issues. This knowledge will be highly usable for educators, school managers and policy makers when tackling, for instance local challenges regarding school-based PA.

The detailed information on project delivery and roll-out at the school level makes it possible to better inform future practice. This study is co-created with and delivered by educators and school managers, which ensures that the findings and experiences are particularly relevant for practice.

There are considerations within the study, which must be taken into account. Firstly, the measurement of psychosocial well-being relies on data generated via survey tools, which can only grasp parts of this complex phenomenon. Secondly, the multi-component design makes it difficult to single out the effectiveness of, for instance, individual PA activities. Thirdly, although 3124 pupils and nine months follow-up are considered a comprehensive design, there is still limited power to detect a real intervention effect. Facilitating and detecting robust improvements in psychosocial well-being among children and youths via PA may very well take years instead of months. Fourthly, the intervention has to be balanced between tailoring the intervention to the individual school and ensuring a certain degree of comparability. Too much tailoring could lead to a non-comparable intervention and too much conformity could entail lower motivation for implementation among key stakeholders.

## Abbreviations

CDP: Competence development program
CG: Coordination group
C-PSPP: Children’s physical self-perception profile
HRQoL: Health-related quality of life
ICC: Intracluster correlation coefficient
KT: Knowledge translation
MWS: Move for well-being in school
PA: Physical activity
PACES: Physical activity enjoyment scale
PASES: Physical activity self-efficacy scale
PDG: Project development group
PE: Physical education
RCT: Randomized controlled trial
RFI: Room for improvement
SDT: Self-determination theory
SPPC: Self-perception profile for children
TAP: Tailored activity program
WHO: World Health Organisation

## Footnotes

1 WHO: Process of translation and adaptation of instruments.
